# Basic Units of Inter-Individual Variation in Resting State Connectomes

**DOI:** 10.1038/s41598-018-38406-5

**Published:** 2019-02-13

**Authors:** Chandra Sripada, Mike Angstadt, Saige Rutherford, Daniel Kessler, Yura Kim, Mike Yee, Elizaveta Levina

**Affiliations:** 1 Department of Psychiatry, University of Michigan, Ann Arbor, MI USA; 2 Department of Statistics, University of Michigan, Ann Arbor, MI USA

## Abstract

Resting state functional connectomes are massive and complex. It is an open question, however, whether connectomes differ across individuals in a correspondingly massive number of ways, or whether most differences take a small number of characteristic forms. We systematically investigated this question and found clear evidence of low-rank structure in which a modest number of connectomic components, around 50–150, account for a sizable portion of inter-individual connectomic variation. This number was convergently arrived at with multiple methods including estimation of intrinsic dimensionality and assessment of reconstruction of out-of-sample data. In addition, we show that these connectomic components enable prediction of a broad array of neurocognitive and clinical symptom variables at levels comparable to a leading method that is trained on the whole connectome. Qualitative observation reveals that these connectomic components exhibit extensive community structure reflecting interrelationships between intrinsic connectivity networks. We provide quantitative validation of this observation using novel stochastic block model-based methods. We propose that these connectivity components form an effective basis set for quantifying and interpreting inter-individual connectomic differences, and for predicting behavioral/clinical phenotypes.

## Introduction

Resting state functional connectomics has emerged as a leading method for mapping the organization of human brain networks^1–5^. In addition, it presents a major opportunity for elucidation of the brain basis of individual differences^6–9^: functional networks are thought to be critical substrates for major neurocognitive and behavioral phenotypes^10–12^, so across-individual differences in network organizations may predict differences in these phenotypes^13,14^. The eventual goal is to refine phenotypic prediction sufficiently that functional connectomes can serve as reliable, objective “biomarkers” of clinically meaningful traits and dimensions^7,15–18^.

Notably, while attempts to utilize functional connectomes for prediction of individual differences are numerous^13,14^, attempts to descriptively assess the nature, kind, and extent of population-wide inter-individual functional connectomic variation remain scarce^19,20^. One important open question concerns the dimensionality of inter-individual variation.

In high dimensional data, there is often substantial dependency in the feature set, and it is often useful for a wide of variety of purposes—computation, interpretation, explanation, and prediction—to identify low-rank structure in the data, i.e., major components that explain a substantial portion of the variation. Over the last 15 years, there has been extensive work in detecting low-rank structure in *intra-individual* across-time variation in the connectome, i.e., the tendency of distributed brain regions to exhibit coherent fluctuations in their BOLD time series^21–23^. This work has culminated in the identification of a small number of intrinsic connectivity networks (ICNs) as major components of intra-individual cross-time variation^2,24,25^. These networks, in turn, have played central roles in recent models and explanations of cognitive capacities and behavioral phenotypes^6,9–12,26^.

Importantly, however, there have not been corresponding systematic attempts to identify low-rank structure in patterns of *inter-individual* variation (but see^27–29^ for limited attempts). This is the question we address in this study. That is, analogous to the intra-individual case, are there major components of inter-individual variation that explain a sizable portion of cross-individual connectomic differences, and that can be effectively harnessed for the purposes of understanding and predicting phenotypes of interest?

In this study, we provide evidence that the answer to this question is yes. Using convergent methods, we show that a modest number of connectivity components, around 50–150, do indeed capture a sizable share of inter-individual differences, and they together constitute a highly effective basis set for phenotypic prediction. Thus, while the resting state connectome is a massive and complex object encompassing tens to hundreds of thousands of connections (depending on the parcellation), differences in a fairly small set of components explain a sizable portion of how any two individuals meaningfully differ.

## Methods

### Subjects and data acquisition

All subjects and data were from the HCP-1200 release^30,31^. All subjects provided informed consent. Subject recruitment procedures and informed consent forms, including consent to share de-identified data, were approved by the Washington University institutional review board, and all research was performed in accordance with relevant guidelines and regulations. Four runs of resting state fMRI data (14.5 minutes each; two runs per day over two days) were acquired on a modified Siemans Skyra 3 T scanner using multiband gradient-echo EPI (TR = 720 ms, TE = 33 ms, flip angle = 52°, multiband acceleration factor = 8, 2 mm isotropic voxels, FOV = 208 × 180 mm, 72 slices, alternating RL/LR phase encode direction). T1 weighted scans were acquired with 3D MPRAGE sequence (TR = 2400 ms, TE = 2.14 ms, TI = 1000 ms, flip angle = 8, 0.7 mm isotropic voxels, FOV = 224 mm, 256 sagittal slices). T2 weighted scans were acquired with a Siemens SPACE sequence (TR = 3200 ms, TE = 565 ms, 0.7 mm isotropic voxels, FOV = 224 mm, 256 sagittal slices).

Subjects were eligible to be included if they had structural T1 and T2 data and had 4 complete resting state fMRI runs (14 m 30 s each; 1206 subjects total in release files, 1003 with full resting state and structural).

### Data preprocessing

Processed volumetric data from the HCP minimal preprocessing pipeline including ICA-FIX denoising were used. Full details of these steps can be found in Glasser^32^ and Salimi-Korshidi^33^. Briefly, T1w and T2w data were corrected for gradient-nonlinearity and readout distortions, inhomogeneity corrected, and registered linearly and non-linearly to MNI space using FSL’s FLIRT and FNIRT. BOLD fMRI data were also gradient-nonlinearity distortion corrected, rigidly realigned to adjust for motion, fieldmap corrected, aligned to the structural images, and then registered to MNI space with the nonlinear warping calculated from the structural images. Then FIX was applied on the data to identify and remove motion and other artifacts in the timeseries. These files were used as a baseline for further processing and analysis (e.g. MNINonLinear/Results/rfMRI_REST1_RL/rfMRI_REST1_RL_hp2000_clean.nii.gz from released HCP data).

Images were smoothed with a 6 mm FWHM Gaussian kernel, and then resampled to 3 mm isotropic resolution. This step as well as the use of the volumetric data, rather than the surface data, were done to allow comparability with other large datasets in ongoing and planned analyses that are not amenable to surface-based processing.

The smoothed images then went through a number of resting state processing steps, including a motion artifact removal steps comparable to the type B (i.e., recommended) stream of Siegel *et al*.^34^. These steps include linear detrending, CompCor to extract and regress out the top 5 principal components of white matter and CSF^35^, bandpass filtering from 0.1–0.01 Hz, and motion scrubbing of frames that exceed a framewise displacement of 0.5 mm. Subjects with more than 10% of frames censored were excluded from further analysis, leaving 966 subjects. A resting state quality control plot^36^ relating motion effects by edge length showed a near zero mean (0.006), low dispersion around the mean (sd 0.06) and absence of a meaningful distance-dependent relationship.

### Connectome generation

We next calculated spatially-averaged time series for each of 264 4.24 mm radius ROIs from the parcellation of Power *et al*.^25^. We then calculated Pearson’s correlation coefficients between each ROI. These were then were transformed using Fisher’s r to z-transformation.

### Train/Test/Retest Split

The 966 subjects after exclusions were divided into three groups. First, 38 subjects who had two separate completed scans were set aside for later test-retest reliability analysis. Of the remaining subjects, 18 did not have complete behavioral data for our analyses so were excluded. Next, 100 unrelated subjects were randomly selected from all unrelated subjects to serve as our held out test set, with the other 810 serving as our training set.

### Estimation of intrinsic dimensionality

In the training dataset, each subject’s connectome was vectorized and concatenated yielding an 810 subjects × 34,716 connections matrix. We estimated the number of intrinsic dimensions of this matrix using two methods.

First, we used a maximum likelihood estimation method based on distance between similar subjects^37^, appropriate for low-dimensional data that is embedded in a high-dimensional space in a complicated, potentially non-linear, fashion. Levina and Bickel^37^ provide a full derivation of the estimator using a Poisson approximation and demonstrate improved performance relative to alternatives in simulated and real data. The method averages over a range of values of *k*, the number of nearest neighbors, from *k*_*1*_ to *k*_*2*_. We used the default values *k*_*1*_ = 10 to *k*_*2*_ = 20 suggested by the original paper.

For comparison, we also applied the method of Choi *et al*.^38^, which aims to provide an upper bound on the number of dimensions with exact type 1 error control. This is a distribution-based method that leverages a post-selection inference framework, extending the work of Taylor, Loftus, and Tibshirani^39^ to the PCA setting.

### Principal component analysis

The subjects *x* connections matrix from the training dataset was next submitted to principal components analysis using the pca function in MATLAB, yielding 809 components ordered by descending eigenvalues.

### Visualizing and assessing low-rank structure

After performing PCA on the training dataset, we converted eigenvalues into percentage variance explained by dividing each eigenvalue by the sum of all eigenvalues, and plotted these percentages from highest to lowest. To assess how much these deviate from what one would expect to find by chance, we constructed an empirical null distribution of percentage variance explained as follows. We represented each subject by their BOLD time series for each ROI, with time series arranged in rows and ROIs corresponding to columns. Time series differed slightly in length across subjects due to motion scrubbing. Thus we calculated the minimum sized time series for any included subject (4320 timepoints) and randomly retained 4320 time points for each subject. Then for each subject *i* and each ROI *j*, we switched the associated time series with ROI *j*’s time series from some other randomly selected subject. This destroyed dependence between ROIs within a subject, while maintaining a realistic data structure in every other way. These shuffled subject matrices were converted to connectomes as before, PCA was applied, and percentage variance explained was obtained for all eigenvalues. This was repeated 1000 times to obtain a distribution of percent variance explained values for leading eigenvalue components. This distribution corresponds to the null hypothesis of no dependence between ROIs and therefore absence of low-rank structure, and can be used to assess whether the data could have appeared to be low rank by sheer chance.

### Assessing out-of-sample reconstruction

We examined the ability of a *k*-sized basis set (consisting of the first *k* PCA components ordered by descending eigenvalues), to reconstruct out-of-sample data, systematically varying the size of *k*. First, a full set of 809 PCA components were learned on the training dataset. Next, for each value of *k* from 1 to 809, we did the following: Using multiple regression, each subject in the held out test dataset was reconstructed as a linear combination of the first *k* components. Goodness of reconstruction was measured by calculating the Pearson’s correlation across edges between actual versus reconstructed connectomes for each subject, and averaging across subjects.

### Assessing phenotypic prediction

#### HCP phenotypic measures

We used a total of 11 phenotypes from the HCP data. Factor analysis, implemented in SPSS 23 (IBM, Armonk, NY), was used to produce two neuropsychological factors from the HCP task data. First, a general executive factor was created based on overall accuracy for three tasks: *n*-back working memory task, relational processing task, and Penn Progressive Matrices task. Factor loadings were 0.81, 0.80, and 0.76 respectively, and the factor accounted for 62.2% of the variance in the variables. A speed of processing variable was created based on three NIH toolbox tasks: processing speed, flanker task, and card sort task (all age-adjusted performance), similar to^40^. Of note, the first of these three tasks is designed to be a measure of processing speed, while the latter two primarily reflect processing speed because for most subjects in the HCP dataset, accuracy is close to ceiling^41^. This variable had loadings of 0.75, 0.81, and 0.82 respectively, and the factor accounted for 63.0% of the variance in the variables. From the Adult Self Report (ASR) instrument^42^, we used three scale-derived summary scores for psychopathology: overall internalizing, overall externalizing, and attention. In addition, from the Neuroticism/Extroversion/Openness Five Factor Inventory instrument^43^, we used the five personality factors: openness to experience, conscientiousness, extroversion, agreeableness, and neuroticism. Finally, we used the Penn Progressive Matrices task by itself as it has been featured in other connectome-based prediction studies of HCP data^44,45^.

In an additional analysis, we used multiple regression to remove a number of potential confounders from each of the 11 phenotypic variables. Following a recent analysis that used HCP data to predict phenotypes^46^, variables regressed from the phenotypes were: age, age squared, mean FD, mean FD squared, gender, brain size (S BrainSeg Vol), brain size squared, and multiband reconstruction algorithm version number (fMRI 3 T ReconVrs). Analyses involving phenotypic prediction were then repeated with the confounder-adjusted phenotypes. Results were broadly similar to the original analyses, and are presented in the Supplement.

#### Brain basis set modeling

To generate predictions of phenotypes from a basis set consisting of *k* components, we used Brain Basis Set (BBS) modeling (similar to the approach introduced in^27^). This approach is similar to principal component regression^47,48^, with an added predictive modeling element. In a training partition, we calculate the expression scores for each of *k* components for each subject by projecting each subject’s connectivity matrix onto each component. We then fit a linear regression model with these expression scores as predictors and the phenotype of interest as the outcome, saving B, the *k* × *1* vector of fitted coefficients, for later use. In a test partition, we again calculate the expression scores for each of the *k* components for each subject. Our predicted phenotype for each test subject is the dot product of B learned from the training partition with the vector of component expression scores for that subject.

#### Identification of prediction plateaus using 10-fold cross-validation

We assessed prediction of HCP phenotypes as a function of the number of basis components used for prediction, in order to identify plateaus where adding additional components does not enhance predictive accuracy. This analysis was performed using a 10-fold cross-validation procedure within the training dataset split described above (to preserve the test dataset for additional analyses described below). On each of the ten folds, we used the training folds to learn new PCA components and then estimated regression coefficients for BBS modeling. We then made predictions for the phenotypes on the held out fold. The correlations between actual phenotype and predicted phenotype on the held out fold were then averaged across the ten folds.

#### BBS comparison with CPM using independent sample validation

To further assess the effectiveness of a low-rank basis set for capturing phenotypic differences in the HCP dataset, we compared the accuracy of phenotypic predictions derived from the 100 component basis set (coupled with BBS modeling) with predictions from an alternative leading method: connectome predictive modeling (CPM)^49^, which has achieved excellent results in a number of studies using diverse phenotypes^44,50–53^. In brief, CPM is first trained with every edge of the connectome to identify edges that are predictive of the phenotype of interest above some prespecified level (e.g., Pearson’s correlation with significance of *p* < 0.01). The sum of weights for these specified edges is then calculated for each training subject and related to actual scores with a linear model. Test subject sums are calculated and multiplied by the beta from the fitted model to generate predicted scores that are correlated with the actual phenotypic scores. CPM treats positively and negatively predictive edges differently, and we focus on the positive edges in the main article, following the typical practice of its authors, and present results for negative edges in the Supplement. We assessed the accuracy of predictions with each method with correlation coefficients between actual and predicted phenotypes.

### Density of parcellation analysis

To assess the robustness of the analysis to parcellations of systematically varying densities, we used the set of parcellations created by Craddock *et al*.^54^. These parcellations (available here: http://ccraddock.github.io/cluster_roi/atlases.html) were produced with a spatially constrained spectral clustering approach that, for preset values of *K*, produces approximately *K* functionally and spatially coherent regions. We utilized parcellations with *K* ranging from 100–900 in intervals of 100. For each parcellation, we repeated the above analyses in order to assess whether our three methods for identifying low-rank structure (assessment of: intrinsic dimensionality, out-of-sample reconstruction, and phenotypic prediction) differed according to parcellation density. Of note, our implementation of the method of Choi *et al*. did not converge for larger parcellations (K > 500) and so we focus on the method of Levina and Bickel for this analysis.

### Assessing community structure

For all 809 components derived from the training dataset, we assessed the presence of community structure corresponding to ICNs from the parcellation of Power *et al*.^25^ by fitting a stochastic block model (SBM)^55^, a well-established generative model for graphs, coupled with a non-parametric testing procedure. For each of the 809 components, we first fix node community assignments according to the Power *et al*. parcellation^25^, and then estimate the parameters of a SBM with these fixed assignments. We replace the Bernoulli distribution assumption on binary edges made by the classical SBM with a normal distribution assumption on edge weights, since we work with Fisher-transformed correlations as edge weights. Once these parameters are estimated, we compute the corresponding profile log-likelihood statistic. We then randomly permute node labels many times, keeping the total number of nodes in each of the communities fixed, re-estimate parameters, and obtain a profile log-likelihood value for an SBM corresponding to permuted node community labels. We then obtain a p-value by comparing the profile log-likelihood for the Power parcellation to the empirical null distribution of profile log-likelihoods, adjusting for multiple comparisons using Bonferroni’s correction to control the Family-Wise Error Rate at α = 0.05. A more detailed description of this procedure is provided in the Supplement.

### Test/Retest reliability

Test-retest reliability was assessed in 38 subjects in the HCP test-retest dataset. Reliability was assessed with the intra-class correlation (ICC) statistic, specifically type (2,1) according to the scheme of Shrout and Fleiss^56^. For each subject, ICC’s were calculated for each individual edge as well as for expression scores for each component in the 100-member basis set. Since aggregating edges can itself improve ICC, we also examined ICC’s for “random” aggregations of edges, created by randomly permuting the columns of each vectorized component. For each component, 1000 randomly permuted components were created in this way. ICC’s for the expressions of these components were calculated, and we report the mean and 95% confidence interval for the permutation-based null distribution.

## Results

### There is convergent evidence for low-rank structure in inter-individual connectomic variation based on three different methods

#### Method 1: Assessing intrinsic dimensionality

**Figure 1:**
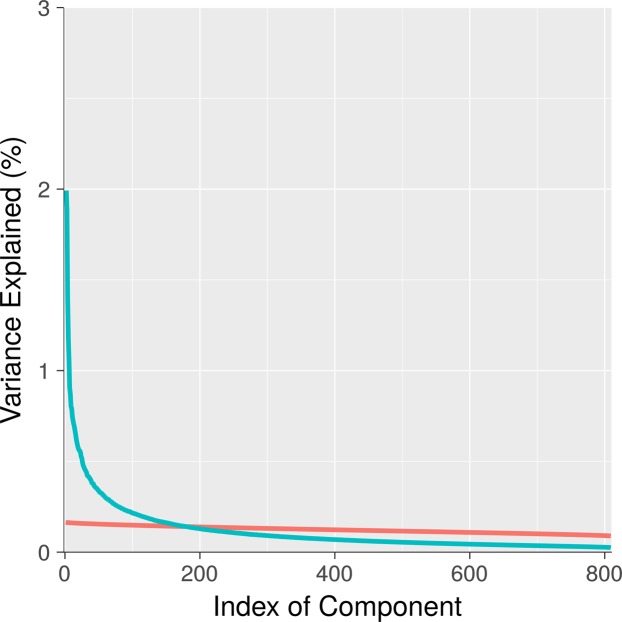
Percent Variance Explained For Components Derived From Actual Versus Random Data. Components are ordered by decreasing percent variance explained. For observed components (blue), percent variance explained of early components is much larger than mean percent variance explained of components derived from random data (red). This pattern is suggestive of substantial low-rank structure in observed connectomes. Of note, the 95% confidence interval is plotted on the red line, but it is too narrow to be visible.

Figure 1 shows the percent variance explained (i.e., eigenvalues) for all 809 components (in blue). Also plotted is mean eigenvalues for 1000 realizations of random data created through permutation methods (in red). This plot provides initial suggestive evidence of significant low-rank structure in the data, indicated by the substantially elevated variance explained by early components derived from observed connectomes relative to components derived from random data.

We next turned to quantitative dimensionality estimation procedures. Applying the maximum likelihood method from Levina and Bickel^37^ yielded an estimated dimensionality of 62. Applying the dimensionality estimation method of Choi *et al*.^38^ found an upper bound of 147 components with α set at 0.05. Importantly, these two results should be seen as complementary and not contradictory, as the Levina and Bickel method estimates the number of components that is *most likely* given the data, while the Choi *et al*. method provides an *upper bound* on the number of components, with statistical control over type 1 errors. Taken together, these results provide strong initial evidence for substantial low-rank structure in cross-individual connectomic variation. In addition, they suggest a plausible range for the number of true dimensions in the data as being somewhere between 50 and 150.

#### Method 2: Assessing out of sample reconstruction

**Figure 2:**
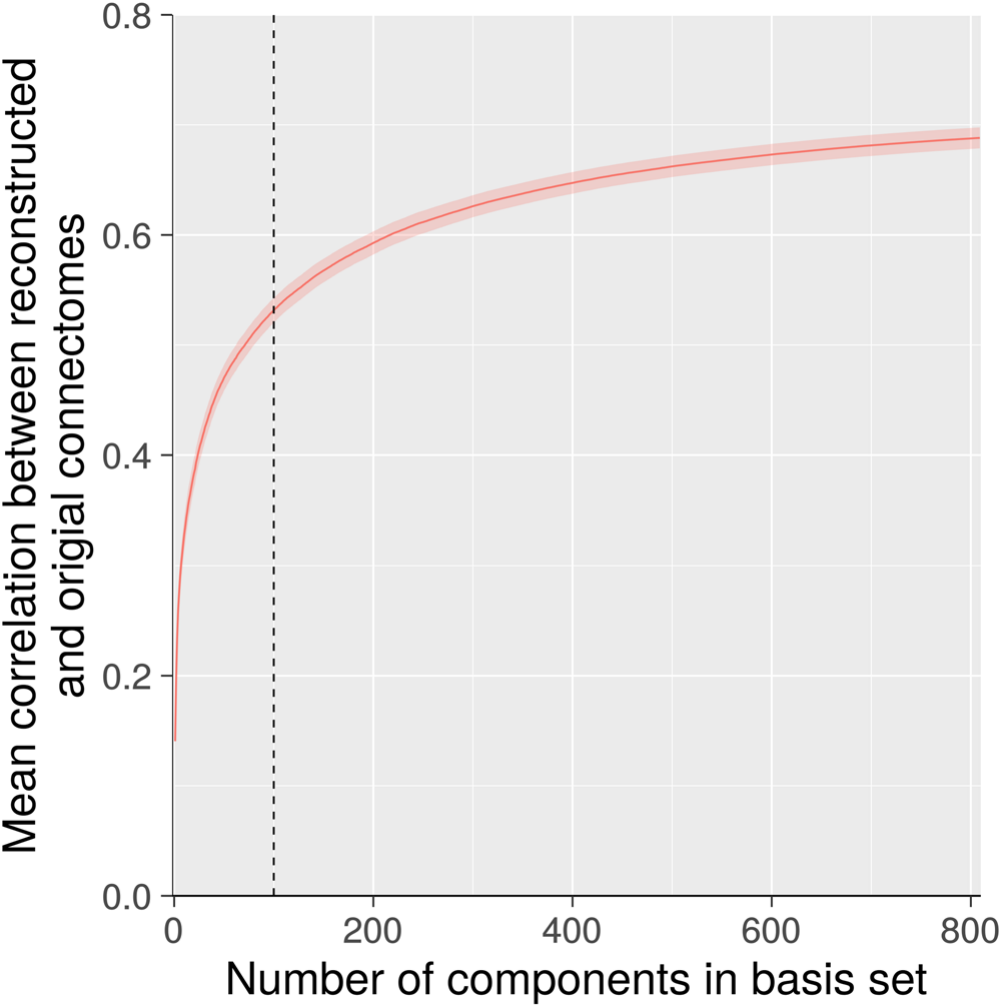
Out of Sample Reconstruction of Connectomes. With 100 components (dashed line), the correlation between actual and reconstructed connectomes is 0.50 (mean correlation across subjects; shading represents 95% confidence interval). Importantly, this correlation is only 0.68 using all 809 components, so a basis set consisting of 100 components achieves roughly three fourths of the “ceiling” correlation that is achievable.

A second method for detecting and quantifying low-rank structure relies on examining the ability of the PCA components to accurately reconstruct connectomes from the independent test dataset, i.e., a sample that was not used to generate the components. Figure 2 shows the Pearson’s correlations between actual test sample connectomes and connectomes reconstructed with a PCA-derived basis set, as a function of the number of components in the basis set. Using all 809 components in the basis set, this correlation was 0.68, and this represents the ceiling correlation that is achievable. With 50, 100, and 150 components, the correlation is 0.47, 0.53, 0.57, respectively. This represents, respectively, 69%, 78%, and 84% of the ceiling obtained with all the components, and it provides additional evidence that a low-rank representation captures a sizable portion of the generalizable variation in the data.

#### Method 3: Assessing predictive accuracy with respect to a broad range of HCP phenotypes

An additional means to assess low-rank structure consists in examining prediction of criterion variables: If a modest sized basis set captures a large portion of cross-individual variation, then it ought to predict a broad range of behavioral and clinical phenotypes (that are plausibly linked to functional connectomic variation) similarly to the full unreduced dataset.

**Figure 3:**
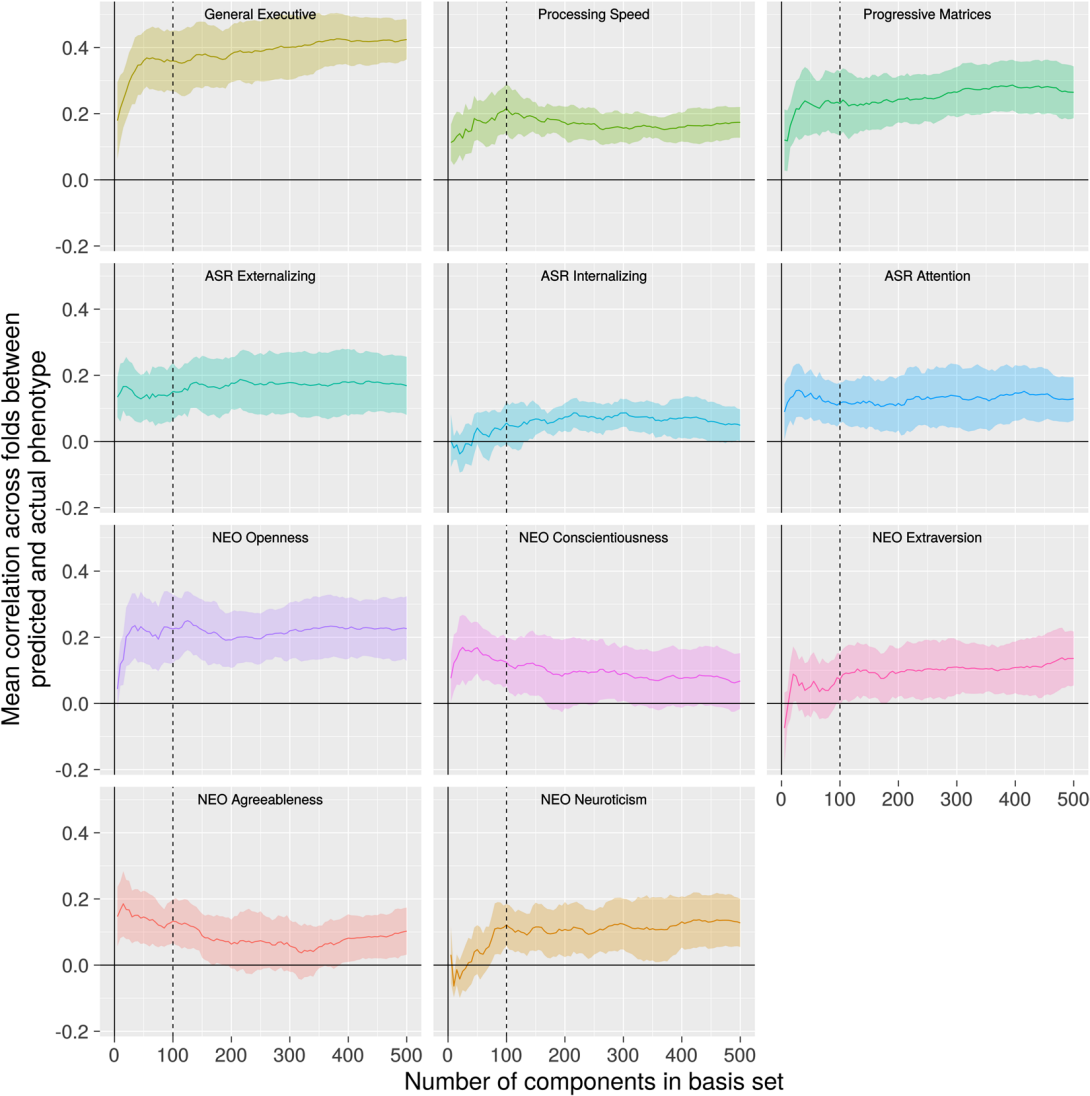
Phenotype Predictive Accuracy as a Function of Basis Set Size. For most phenotypes, there is a plateau after 50 to 100 components (dotted line) after which adding further components to the prediction model basis set does not appreciably improve performance. Shading reflects 95% confidence interval based on cross-validation error.

For each of 11 phenotypes, we used the BBS modeling method to make predictions of phenotype values for each subject based on connectomic component expression scores. We applied BBS to the 810 subjects in the training dataset in a 10-fold cross validation procedure. As shown in Fig. 3, there is a noticeable plateau at around 50–100 components for most of the phenotypes: Adding further components to the basis set beyond this number does not appreciably increase accuracy of phenotypic prediction. Table S1 shows the correlations between predicted and actual phenotypes across three basis set sizes: 50, 100, and 150 components. All three basis sets perform similarly, though there is a slight advantage for the 100-component basis set, especially with regard to the processing speed factor.

To further assess the performance of a modest sized basis set in predicting phenotypes of interest, we compared performance with CPM, a leading alternative method for phenotypic prediction that is trained on the whole connectome^49^. Since the 100-component basis set performed slightly better than the others in cross-validation within the training dataset, we focused on this basis set for the BBS comparison with CPM.

For each of the 11 phenotypes, we trained both methods in the training dataset of 810 subjects. We then tested accuracy of phenotypic prediction in the held out test dataset of 100 subjects. Results showed that performance of BBS was comparable to or better than CPM on all 11 phenotypes (comparable to CPM on 8 phenotypes and better than CPM on the other 3 phenotypes; Table 1).

**Table 1:** Pearson’s correlations between actual and predicted phenotypes for two different predictive modeling approaches.

Phenotype	BBS	CPM
General Executive	0.44*	0.42*
Processing Speed	0.39*	0.23*
Penn Progressive Matrices	0.30*	0.32*
ASR Externalizing	0.24*	0.03
ASR Internalizing	0.20*	0.04
ASR Attention	0.21*	0.00
NEO-Openness	0.18	0.11
NEO-Conscientiousness	0.19	0.15
NEO-Extroversion	0.13	0.04
NEO-Agreeableness	0.19	0.10
NEO-Neuroticism	0.00	0.05

Results are for predictive models trained on a train dataset and tested on an independent test dataset. *BBS* = *Brain Basis Set Modeling (with 100 component basis set); CPM* = *Connectome Predictive Modeling*^49^. *Correlation is significantly different than zero at *p* < 0.05, BOLDED TEXT = correlation is significantly greater than that observed with comparison method at *p* < 0.05.

#### Robustness to parcellation density

**Figure 4:**
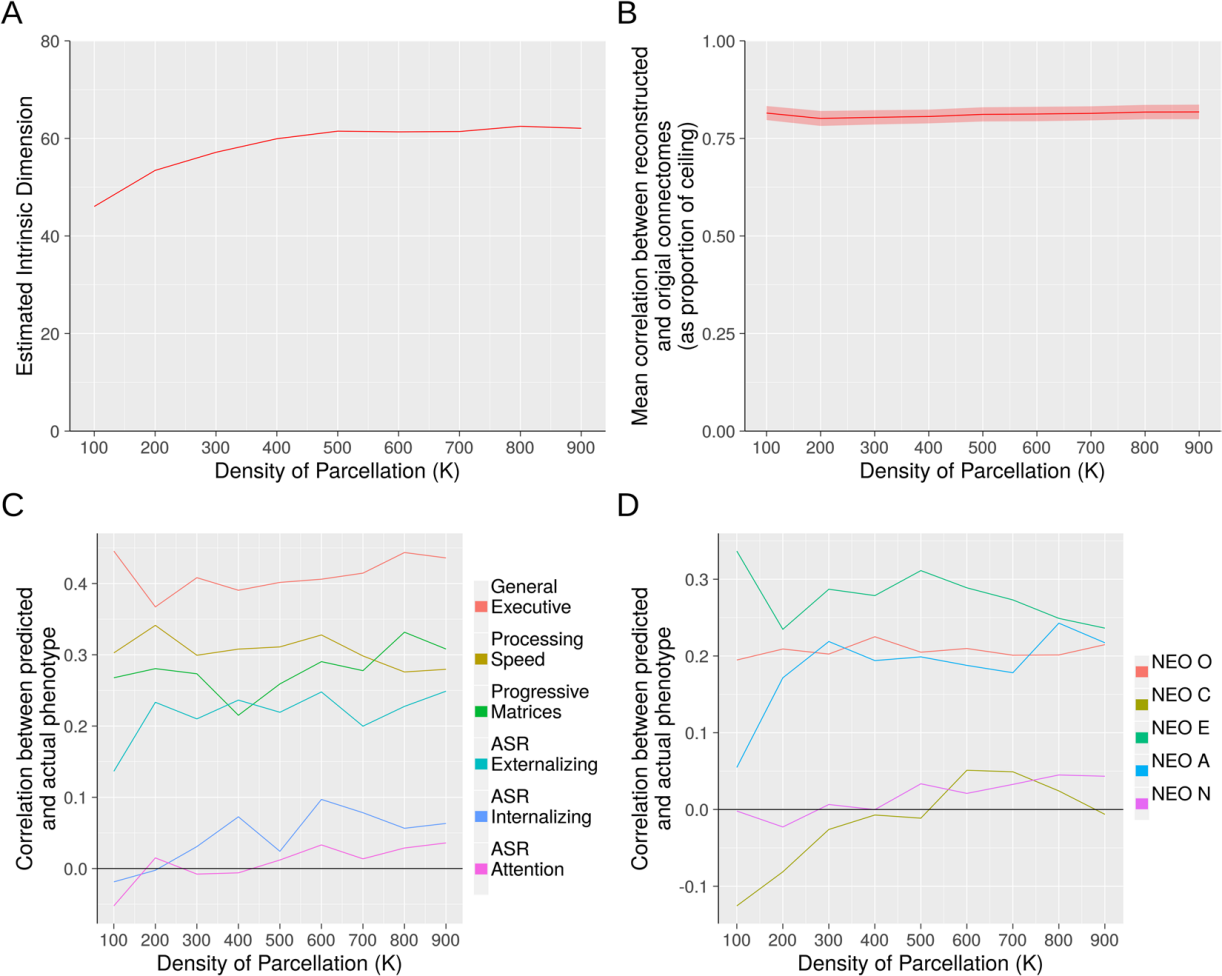
Assessing Robustness to Parcellation Density. Three methods for identifying low-rank structure yielded stable results across parcellations of varying density. Panel (A) Estimation of intrinsic dimensionality with the method of Levina and Bickel^37^. Panel (B) Out-of-sample reconstruction. Panels (C,D) Predictive accuracy with respect to 11 HCP phenotypes. Panels B through D used a 100-component basis set.

We next examined the robustness of the preceding three analyses to parcellations of varying densities. We used Craddock *et al*.’s parcellations derived from a spectral clustering algorithm with *K*, the prespecified number of parcels, set from 100 to 900 in increments of 100. While there were some differences observed with the most sparse parcellation (*K* = 100), for all analyses in which *K* exceeded 200, the results were highly stable and broadly similar to what we observed with the Power parcellation with 264 ROIs (Fig. 4).

### There is substantial network structure in components of inter-individual connectomic variation

**Figure 5:**
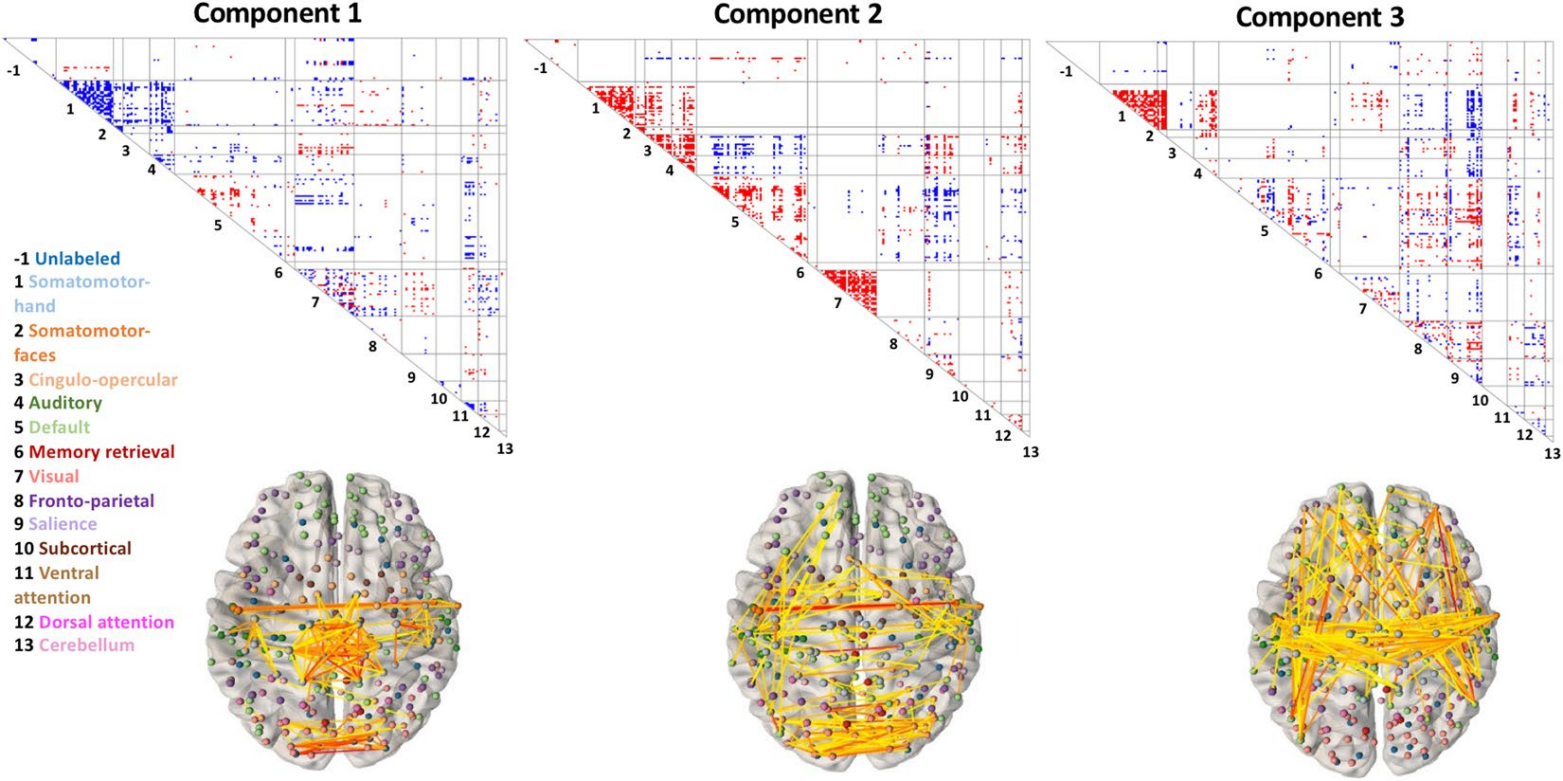
Components 1, 2, and 3. The first three components of inter-individual connectomic variation are displayed, with nodes organized by membership in 13 ICNs according to assignments of Power *et al*.^25^. The boundaries of ICNs are determined from a strictly intra-individual phenomenon: coherence of the BOLD time series within a person across time. It is notable, then, that inter-individual connectomic differences clearly involve substantial ICN structure (which we further corroborate utilizing a novel quantitative approach based on stochastic block modeling). 1 = Somatomotor-hand; 2 = Somatomotor-faces; 3 = Cingulo-opercular; 4 = Auditory; 5 = Default; 6 = Memory retrieval; 7 = Visual; 8 = Fronto-parietal; 9 = Salience; 10 = Subcortical; 11 = Ventral Attention; 12 = Dorsal Attention; 13 = Cerebellum.

We next turn to characterizing connectivity patterns in the components themselves. Figure 5, panels A through C, shows the first three components with nodes organized by membership in ICN communities (e.g., default network, fronto-parietal network, etc.) according the node assignments of Power *et al*.^25^. Qualitatively, these components appear to exhibit prominent ICN structure: the lines on these figures, which represent boundaries of ICN-ICN interrelationships, appear to be highly informative for characterizing connectivity patterns in the components.

**Figure 6:**
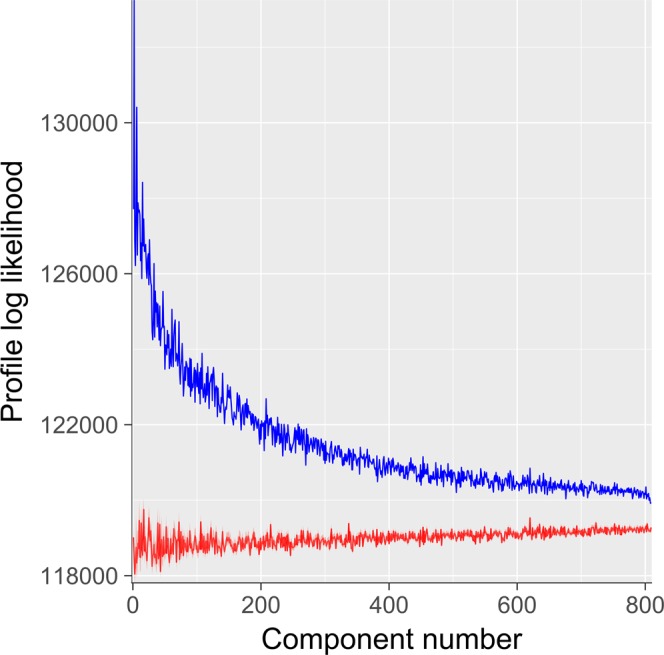
Profile Log-Likelihood of ICN-Based Community Structure in Each Component. This statistic serves to quantify presence of ICN structure in each component using a stochastic block model (SBM) framework. The blue trace is the profile log-likelihood from the SBM according to the community assignments given in Power *et al*.^25^. Note that ICN structure is most prominent in early (high variance explained) components. The red trace is the median profile log-likelihood across many random shufflings of the community assignments and serves as a null distribution. Of note, the 95% confidence interval of the null distribution is plotted on the red line, but it is very narrow and not easily visible.

To quantitatively assess the presence of ICN-based community structure in these components, we utilized an SBM-based method as described in *Methods* coupled with permutation tests for statistical significance. We found that for all 809 components, the observed components’ connectivity patterns are highly statistically significantly more likely under Power ICN community assignments than alternative randomly shuffled assignments (permutation-based p-values for all components survive Bonferroni correction for 809 tests with α = 0.05). Additionally, as a descriptive follow up to quantify the extent of network structure in the components, we investigated how, for each component, the profile log-likelihood corresponding to the Power *et al*. parcellation differed from the empirical profile log-likelihood across the permutations (see Fig. 6). This analysis suggests that while ICN structure is significantly present in all components, such structure is most prominent in early components and plateaus substantially around component 100 to 200.

### Components of inter-individual connectomic variation exhibit significantly higher test-retest reliability than individual edges of the connectome

The preceding analyses suggest that a modest-sized basis set is sufficient to quantify cross-individual variation across the entire connectome, especially the meaningful (i.e., phenotypically predictive) aspects of this variation. A further question concerns the stability of the basis set—or more specifically, subjects’ component expression scores—across scanning sessions.

To address this question, we examined the ICCs of component expression scores in the 38 HCP test-retest subjects. Components were generated in the full training dataset and assessed across the two scanning sessions of the test-retest dataset, which was not used to generate the components. The mean ICC for individual edges was 0.54, similar to values seen in previous studies^57^. In contrast, the ICC for the components of cross-individual variation were notably higher. Focusing on the 100-component basis set, which performed well in phenotypic prediction, the mean ICC was 0.77.

Some of this improvement might be due to aggregation itself, as aggregates tend to be more stable than the elements that are aggregated. To test this possibility, we calculated the mean ICC for random permutations of these 100 components (1000 permutations of each component). Mean ICC for permuted components was 0.6520 (95% confidence interval 0.6514 to 0.6525), so the boost in ICC seen in the actually observed 100 components is significantly and substantially over and above what can be explained by simply aggregating random collections of edges.

## Discussion

In resting state fMRI, the presence of low-rank structure in *intra*-individual variation is well known: a small set of units—ICNs such as DMN and FPN—account for a sizable portion of variation in the BOLD signal across time within a scanning session. In this study, we extend the search for useful low-rank structure to *inter*-individual connectomic variation. We found convergent evidence that a modest number of components, somewhere between 50 and 150, capture a sizable share of how the resting state functional connectomes of any two healthy adults differ. Moreover, we found these components exhibit high levels of network community structure, aiding interpretability, and they have very good test-retest reliability. We propose that the connectivity components identified in this study form an effective basis set for quantifying and interpreting systematic inter-individual connectomic differences and for predicting behavioral and clinical phenotypes.

### The components of inter-individual connectomic variation reflect ICN structure

An interesting feature of the connectomic components that emerged in this study is that they strongly reflect ICN structure. ICN boundaries are determined from a strictly intra-individual phenomenon: coherence of the resting state BOLD time series across regions within a person during a scanning session^24,25,58^. From a statistical point of view, the presence of ICN structure in the BOLD time series makes the identification of ICN structure in our components (which reflect across individual connectivity differences) somewhat more likely, but it is by no means guaranteed—the set of edges that make individuals different could also have crossed ICN boundaries in complex ways. Thus this study adds new knowledge by demonstrating that ICNs are not only a major unit of within-individual differences in BOLD signal over time, but also between-individual differences in functional connectomes.

The finding that there is extensive ICN structure in these components jointly helps to illuminate two issues. First, it helps to explain why we were successful in finding low-rank structure in the first place. Second, it potentially illuminates the mechanisms by which the inter-individual differences we observed arose. Both of these points warrant elaboration.

There is growing understanding of the maturational trajectories of large-scale ICNs and principles by which they take shape. Resting state imaging studies in fetuses suggest at least some important ICNs are in an immature state in the fetal brain with weak intra-network connectivity and low levels of network separation^59–61^. Over the course of childhood to early adolescence, major changes occur: integration of connections within ICNs^62,63^, segregation of default mode network from attention/control networks^27,64,65^, and cross-modal linkages in which structural connections co-develop with functional connections^66–69^. Importantly, there are inter-individual differences in how these developmental changes in ICN-ICN interconnections unfold^27,28,70,71^.

The overall picture, then, involves highly complex and choreographed developmental processes that shape large populations of interconnections between ICNs. This picture is well suited for explaining why we observed significant low-rank structure in inter-individual variation in connectomes, as such structure necessarily exists if individuals systematically differ at large aggregates of connections. In addition, the model explains why the connectomic components themselves exhibit extensive ICN structure, as the presence of such structure naturally follows if the generative processes that produce inter-individual connectomic differences impart aggregate intra- and inter-ICN alterations.

In short, then, we propose that adult inter-individual connectomic variation—especially the meaningful subset of this variation that is relevant to explaining neurocognitive and behavioral phenotypes—importantly reflects the legacy of inter-individual differences in ICN development. This hypothesis invites detailed future investigation, ideally in longitudinal datasets that permit precise quantification of ICN maturational trajectories as well as adult connectomic variation.

### Success at Phenotypic Prediction and Test-Retest Reliability

The Brain Basis Set (BBS) modeling approach leverages a modest number of components of inter-individual variation—in this study we focused on a 100-component basis set. Yet we found this method predicts HCP phenotypic variables (such as executive functioning, processing speed, and externalizing) just as well, or in some cases better than, Connectome Predictive Modeling (CPM), an alternative highly successful method that is trained on every edge of the connectome^49^. The most likely explanation for this result is that systematic connectomic differences across individuals really do have substantial low-rank structure. Thus restricting one’s predictor set to a modest number of connectomic components, which is sufficient to capture this structure, yields strong phenotypic prediction.

An additional complementary explanation emphasizes the issue of signal-to-noise ratio and test-retest reliability. While the inter-session test-retest reliability of individual edges of the resting state functional connectome has been found to be only fair^55,57,72^, the connectomic components identified in this study exhibit substantially better reliabilities. This improvement arises, most likely, because components that explain a large portion of inter-individual connectomic variation are more likely to be latching onto “real” brain differences, i.e., stable cross-individual differences that genuinely exist in nature. In contrast, connectivity features that explain only a tiny portion of inter-individual variation have a greater probability of reflecting noise, which, by definition, lacks test-retest reliability. It follows that restricting analysis to a modest number of high-variance-explained components can boost the signal-to-noise ratio of the included predictors, contributing to better prediction of unseen data (see^73^ for a related argument).

### Uniqueness of the Basis Set

Our primary result concerns the size of the basis set needed to capture meaningful inter-individual connectomic differences. Resting state connectomes, due to their massive size, allow for correspondingly massive variability: individuals could potentially differ in countless ways across tens of thousands of connections. We have shown, however, that actual inter-individual variability is far more limited and most of it is accounted for by a modest-sized basis set of roughly 50–150 components.

We wish to emphasize that with respect to representing the subspace of variation, the connectomic components we identified are not unique. These components are the basis of a subspace, and any rotation that preserves their linear independence will result in a new basis that spans the exact same subspace. There is thus some flexibility in choosing the components with which to characterize the relevant subspace. Ultimately, the choice of which components to utilize must be guided by consilience with broader theory: a basis set should be preferred to the extent that the components that comprise it align with known neurobiological mechanisms and processes. In this context, it bears notice that the PCA-derived components that emerged in this study do exhibit a number of neurobiologically interesting properties. High-variance-explained components, in particular, disproportionately contribute to phenotypic prediction (Fig. 3), and they exhibit higher levels of ICN structure (Fig. 6). This provides initial evidence that that the specific components found by PCA could potentially have neurobiological meaning.

### Implications for connectomic statistical analysis

Our results have broader implications for methods of statistical analyses of connectomes, especially methods aimed at predicting phenotypic differences across individuals and between groups^74,75^. A persistent challenge in individual differences neuroimaging research has been the sheer size of functional connectomes^76^. This sometimes forces researchers to either focus on a small set of “connections of interest” or else undertake a whole connectome statistical search and pay a substantial price in terms of multiple comparisons correction. Our results suggest that the tradeoffs need not be so stark. There is a massive amount of dependence among edges in connectomes across individuals. Thus a basis set with a modest number of components allows researchers interested in individual differences to undertake whole-connectome inquiry while dramatically reducing the multiple comparison cost.

More broadly, there is a pressing need to leverage prior knowledge about the nature, kind, and extent of inter-individual variation in functional connectomes to further guide and constrain statistical models in neuroimaging individual differences research. Our observation of extensive low-rank structure, i.e., a modest number of components account for a sizable portion of cross-individual differences, represents one kind of prior knowledge. Our observation of prominent ICN structure within these components is also highly relevant in this context. Future studies should leverage this observation, for example using block structure-based regularization, to inform and constrain statistical models of inter-individual differences and thereby increase the chances of robust out-of-sample generalization.

In sum, in this study, we identified a parsimonious basis set for inter-individual differences in resting state functional connectomes, one that facilitates interpretation of connectomic differences and prediction of phenotypes of interest. Our results invite further research into the neurodevelopmental processes that shape ICNs, which could help to explain why adult inter-individual connectomic differences take a modest number of characteristic forms.

## Supplementary information

Supplemental Information

## Data Availability

The datasets generated during and/or analysed during the current study are available in the SripadaLab repository: https://github.com/SripadaLab/.
