# Supplementary material for: Basic Units of Inter-Individual Variation in Resting State Connectomes
Source: Sci Rep. 2019 Feb 13;9:1900. doi: 10.1038/s41598-018-38406-5 (PMC6374507; doi:10.1038/s41598-018-38406-5)
Supplement: Supplementary file 1 — Supplemental Information [file 41598_2018_38406_MOESM1_ESM.pdf]

Supplementary Methods

Basic Units of Inter-Individual Variation in Resting State Connectomes

Chandra Sripada[1], Mike Angstadt[1], Saige Rutherford[1], Daniel Kessler[1,2], Yura Kim[2], Mike Yee[1], and Elizaveta Levina[2]

[1]Department of Psychiatry, University of Michigan, Ann Arbor, MI
[2]Department of Statistics, University of Michigan, Ann Arbor, MI

1 Details Regarding Assessment of Community Structure

1.1 The Stochastic Block Model

The SBM [1] is a well-established generative model for networks with communities. Under the SBM, each of the n nodes is independently assigned to one of K communities, with probability of assignment to community k given by π_k, $\sum_{i=1}^K \pi_k = 1$. Given a realization of the community assignments vector c, where c_i is the community label of node i, the SBM generates edge weights A_{ij} between nodes i and j independently, from a distribution depending only on the community labels c_i and c_j. If the distribution is parameterized by a parameter θ_{c_i,c_j}, the distribution of the entire network is determined by the set of parameters θ_{kl}, $k, l = 1, \ldots, K$, with $\theta_{kl} = \theta_{lk}$ if the network is symmetric, as it is in our case. In the classical formulation of the SBM, the adjacency matrix is assumed to be binary, in which case the distribution of A_{ij} is Bernoulli and $\theta_{kl} = P(A_{ij} = 1|c_i = k, c_j = l)$. In our case, because we work with weighted matrices and the weights are Fisher-transformed correlations, we model the distribution of A_{ij} as normal, determined by parameters $\theta_{kl} = (\mu_{kl}, \sigma^2_{kl})$.

1.2 Calculating profile likelihood under the SBM

In our setting, we have an a priori community membership as given by the Power *et al.* parcellation [2]. The log-likelihood of the observed weights for a given community assignment, c, is given by

$$
\begin{aligned}
\log \mathcal{L}(\theta, \pi|A) &= \sum_{k=1}^{K} n_k \log(\pi_k) + \sum_{i=1}^{n} \sum_{j=1}^{i-1} \log f(A_{ij}; \theta_{c_i,c_j}), \\
&= \sum_{k=1}^{K} n_k \log(\pi_k) + \sum_{i=1}^{n} \sum_{j=1}^{i-1} \left[-\frac{1}{2} \log(2\pi) - \log \sigma_{c_i,c_j} - \frac{(A_{ij} - \mu_{c_i,c_j})^2}{2\sigma^2_{c_i,c_j}}, \right]
\end{aligned}
$$

where n_k is the number of nodes in community k, and $f(\cdot; \theta_{kl})$ is the probability density function of $N(\mu_{kl}, \sigma^2_{kl})$.

Maximizing the likelihood of the SBM over community assignments is an NP-hard problem, but for a given c, maximizing over π and θ is easy and there is a closed form solution. Let S_{kl} denote the set of node pairs connecting community k to community l, $S_{kl} = \{i < j : c_i = k, c_j = l\}$, and let $n_{kl} = |S_{kl}|$ denote the number of such pairs. Then the maximum likelihood estimates of parameters for a given c are

$$\hat{\pi}_k = \frac{n_k}{n} \ ,$$
$$\hat{\mu}_{kl} = \frac{1}{n_{kl}} \sum_{(i,j)\in S_{kl}} A_{ij} \ ,$$
$$\hat{\sigma}^2_{kl} = \frac{1}{n_{kl}} \sum_{(i,j)\in S_{kl}} (A_{ij} - \hat{\mu}_{kl})^2 \ ,$$

the usual MLEs under the normal distribution. Plugging in these values into the profile likelihood gives the maximized profile likelihood, which we use as the test statistic.

To carry out the test, we need to compare the value of the observed profile log-likelihood, $\hat{l}$, to the distribution of profile log-likelihoods under the null hypothesis of no community structure in the data. We obtain this distribution empirically, shuffling the labels of the given parcellation c randomly and recomputing the profile log-likelihood in the same way, m = 20,000 times in total, to obtain the values l_j, $j = 1, \ldots, m$. Finally, we estimated empirically the probability that a profile log-likelihood L sampled from this null distribution will exceed $\hat{l}$, as

$$P(L \geq \hat{l}) = \max\left(\frac{1}{m}, \ \frac{1}{m} \sum_{i=1}^{m} I(l_i \ \geq \hat{l})\right),$$

where I is the indicator function.

Note that permutation of the labels does not change the number of nodes in each community, so the terms involving $\hat{\pi}_k$'s can omitted.

This procedure is repeated for each of the 809 components of interest, and the resulting 809 p-values are Bonferroni-corrected for multiple comparisons. The number of permutations was selected such that it would be mathematically possible to achieve Bonferroni-corrected significance at $\alpha = .05$.

In addition, for each component we retained both the profile log-likelihood under the Power *et al.* parcellation [2] and the empirical profile log-likelihood across the m shufflings, and plotted these as a function of the component number (see Figure 6). Because of the use of logs, the ratio of likelihoods is proportional to the difference of log-likelihoods, and one may descriptively interpret the "gap" between the two traces as some indication of the magnitude of the divergence from the null.

References

[1] Paul W. Holland, Kathryn Blackmond Laskey, and Samuel Leinhardt. "Stochastic blockmodels: First steps". In: *Social Networks* 5.2 (June 1983), pp. 109–137. ISSN: 0378-8733. DOI: 10.1016/0378-8733(83)90021-7. URL: http://www.sciencedirect.com/science/article/pii/0378873383900217 (visited on 03/26/2018).

[2] Jonathan D. Power et al. "Functional Network Organization of the Human Brain". In: *Neuron* 72.4 (Nov. 2011), pp. 665–678. ISSN: 0896-6273. DOI: 10.1016/j.neuron.2011.09.006. URL: http://www.sciencedirect.com/science/article/pii/S0896627311007926 (visited on 07/02/2013).
